# Mindful Attention Awareness and Residual Mood and Anxiety Symptoms in Remitted Patients With Bipolar I Disorder

**DOI:** 10.31083/AP46029

**Published:** 2025-08-25

**Authors:** Selin Alkan, Habib Erensoy, Tonguc Demir Berkol

**Affiliations:** ^1^Department of Psychiatry, Ezine State Hospital, 17600 Canakkale, Turkey; ^2^Department of Psychiatry, Uskudar University, Medical Faculty, 34768 Istanbul, Turkey; ^3^Department of Psychiatry, Bakirkoy Research and Training Hospital for Psychiatry, Neurology and Neurosurgery, 34147 Istanbul, Turkey

**Keywords:** anxiety, bipolar disorder, depression, mindfulness

## Abstract

**Objective:**

In bipolar disorder, residual mood symptoms often persist even during the euthymic period, impairing functionality in 30–60% of patients in clinical remission. Addressing residual symptoms is critical as they are linked to reduced functionality and subjective wellbeing. This cross-sectional study aimed to determine the relationship between mindful attention awareness (MASS) and residual symptom severity in bipolar I disorder.

**Methods:**

This study included 100 patients with bipolar I disorder (BD-I) in remission, recruited consecutively from outpatient clinics between December, 2019 and March, 2020. The patients were evaluated using the sociodemographic data form, Hamilton Depression Rating Scale (HAM-D), Young Mania Rating Scale (YMRS), and Mindful Attention Awareness Scale (MAAS).

**Results:**

As the MAAS of the patients increases, their depressive symptoms decrease (*p* < 0.001). As the MAAS of the patients increases, their anxiety symptoms decrease (*p* < 0.001). No statistically significant relationship was found between the mindful attention awareness of the patients and their manic symptoms (*p* = 0.161). Two variables (HAM-D and Hamilton Anxiety Rating Scale Scores (HAM-A)) in the multiple linear regression model explain 21.8% of the change in MAAS score.

**Conclusion:**

Residual depressive and anxiety symptoms are predictive of lower MAAS in bipolar I disorder. Incorporating strategies to manage residual mood symptoms and improve mindfulness may enhance functionality and facilitate complete remission. However, given the cross-sectional design and the lack of a control group, causal inferences cannot be made. Further longitudinal and interventional studies are needed to explore the efficacy of mindfulness-based approaches in BD-I and determine whether the observed associations are specific to this disorder.

## Main Points

∙ Mindful attention awareness is significantly reduced in patients with higher residual depressive and anxiety symptoms in bipolar I disorder.

∙ Residual manic symptoms do not show a significant association with mindful attention awareness.

∙ Depressive and anxiety symptoms are primary predictors of mindful attention awareness, explaining nearly 24.8% of its variance.

∙ Lower mindful attention awareness is associated with impaired functionality in patients with bipolar I disorder.

∙ The findings suggest that mindfulness-based interventions could effectively target residual depressive and anxiety symptoms to improve remission outcomes.

## 1. Introduction

Bipolar disorder (BD) is a chronic psychiatric condition characterized by recurring cycles of mania, hypomania, and depression, significantly affecting patients’ quality of life, cognitive abilities, and socio-occupational functioning [1]. Its lifetime prevalence is between 1–5% and it usually starts in the 20s [2]. It negatively affects the life of the person and their relatives due to reasons such as social and professional functionality impairment, suicide risk, and frequent recurrence and causes disability [3]. Comorbidity is common, with anxiety disorders and substance use disorders frequently co-occurring [4].

Despite advancements in treatment, a significant proportion of individuals with BD experience chronic symptoms and impaired social and professional functioning, even during periods of clinical remission [5, 6]. Residual symptoms, including subsyndromal depressive and manic features, can persist in the euthymic phase, contributing to deficits in cognitive functions such as verbal fluency and planning abilities [7]. Studies indicate that 30–60% of patients in clinical remission report reduced functionality, highlighting the persistence of residual symptoms as a critical treatment target [8, 9]. These residual symptoms are strongly associated with diminished psychosocial well-being and increased relapse risk, highlighting a need for improved treatment strategies [9].

Mindful attention awareness, a core component of mindfulness, is defined as the non-judgmental acceptance of present moment experiences, encompassing environmental, emotional, and physical states, without being swayed by past experiences or future expectations [10]. It involves cultivating a skill that allows individuals to respond to present experiences with less reactivity. This includes reduced emotional impulsivity and decreased rumination. By fostering a sense of interconnectedness with all experiences, mindful attention awareness helps reduce suffering and enhance well-being. This state of awareness is characterized by being fully present, without dwelling on the past or anticipating the future [10, 11]. Mindful attention awareness is a learnable skill, varying in degree among individuals and capable of improvement through practice [11]. It diminishes when individuals act impulsively or react to unexamined feelings and perceptions, losing focus on their immediate surroundings. The essence of mindful attention awareness is best understood through direct experience [11].

To further elaborate on the concept of mindful attention awareness, several key aspects should be highlighted. Mindfulness is defined as “paying attention in a particular way: on purpose, in the present moment, and non-judgmentally”. It involves consciously noticing thoughts and feelings without judging them or trying to change them in any way, while being aware of your breath or body [12]. Mindfulness is defined as a person’s non-judgmental focus of attention and acceptance of the experiences they are having at that moment. With the ability to focus on the present, one can become aware of what is happening here and now, away from the anxiety, fear, and distress caused by past negative experiences and uncertainties and expectations about the future [13]. In more detail, the concept of mindfulness means the acceptance of one’s non-judgmental thoughts and physical awareness of that moment, without being influenced by past or planned experiences and emotions, and the acceptance of the entire environmental and emotional state in which one finds oneself [10, 14].

Mindfulness is a skill that allows one to react less to what is happening right now. It is a way of being intertwined with all positive, negative, and neutral experiences, thus reducing pain and increasing well-being. Mindfulness is about waking up and being aware of what is happening right now. When one is consciously aware, attention is not preoccupied with the past and the future; one does not judge or deny what is in the present; one is entirely in the present [15]. Mindfulness is a skill that can be learned, found in every human being to a greater or lesser extent, but can be improved [11]. Mindfulness disappears when an individual starts to act with their own impulses and unexamined feelings and perceptions, without paying attention to their surroundings intensely [16]. It is stated that mindfulness cannot be understood conceptually but only when it is directly experienced, that is, only when it emerges [17].

Given the potential benefits of cultivating mindfulness, it is important to understand how mindfulness practices can impact various aspects of our being. Mindfulness exercises have been shown to positively influence various physiological and psychological processes, including brain function, autonomic nervous system activity, stress hormone levels, immune system response, and health-related behaviors such as eating, sleeping, and substance use [18]. Regular mindfulness practice supports the volumetric and functional balance of the amygdala, reducing stress reactivity, and increases gray matter density in the hippocampus, enhancing learning and memory [19]. Furthermore, mindful attention awareness has been suggested to mitigate cellular aging by bolstering stress resilience [20].

While mindfulness-based interventions, such as Mindfulness-Based Cognitive Therapy (MBCT), have shown promise in reducing depressive and anxiety symptoms in BD [21, 22], findings on their effectiveness vary [23]. Notably, studies have consistently reported no significant changes in mania scores following MBCT in euthymic bipolar patients [22, 24]. Moreover, there is a relative paucity of research specifically examining the relationship between mindful attention awareness and residual symptoms in bipolar I disorder (BD-I). Although improvement in residual depressive symptoms without manic shift has been reported with MBCT [25], the precise mechanisms and predictors of this improvement, particularly concerning mindful attention awareness, remain unclear. Crucially, the role of mindful attention awareness in mitigating residual symptoms, and thereby enhancing functionality and subjective well-being in bipolar I disorder, requires further investigation.

Understanding the relationship between mindful attention awareness and residual symptoms could inform the development of targeted interventions to improve the quality of life for individuals with bipolar I disorder (BD-I). Therefore, the aim of our study is to determine the relationship between mindful attention awareness and residual symptom severity in bipolar I disorder. We hypothesize that higher levels of mindful attention awareness in patients with bipolar I disorder in remission will be associated with lower residual depressive and anxiety symptoms, while residual manic symptoms will remain unaffected.

## 2. Subjects and Methods

This study was designed as a cross-sectional analysis and included patients recruited from the outpatient units of Bakirkoy Mental Health and Neurological Diseases Training and Research Hospital. A total of 100 patients diagnosed with BD-I according to the Diagnostic and Statistical Manual of Mental Disorders, Fifth Edition (DSM-5) diagnostic criteria were consecutively enrolled between December 2019 and March 2020. The study was approved by the local ethics committee, and all participants provided written informed consent before participation.

### 2.1 Participants

To be eligible for the study, participants had to be between 18 and 65 years old, have a diagnosis of BD-I confirmed by a psychiatrist using the Structured Clinical Interview for DSM-5 (SCID-5), and be in clinical remission, defined as a Young Mania Rating Scale (YMRS) score <13 and a Hamilton Depression Rating Scale (HAM-D) score <8 at the time of assessment.

Patients were excluded if they had current psychotic symptoms, a diagnosis of mania, hypomania, or major depressive episode at the time of the study, or any other psychiatric comorbidities, including anxiety disorders, obsessive-compulsive disorder (OCD), post-traumatic stress disorder (PTSD), or personality disorders. To minimize confounding effects, patients with a history of substance abuse or dependence (excluding nicotine) were also excluded. However, patients with a past history of alcohol use disorder occurring more than five years prior were recorded, and these data are included in the demographic table. Additional exclusion criteria included neurological conditions, a history of head trauma, and prior electroconvulsive therapy (ECT) within the past six months. Furthermore, pregnant or lactating women were not eligible to participate.

### 2.2 Sample Size, Recruitment, and Assessment

A power analysis was conducted to determine the required sample size for the study. Based on Cohen’s guidelines for effect sizes [26], we aimed to detect a moderate effect size (r = 0.3) for the correlations between the Mindful Attention Awareness Scale (MAAS) and other clinical scales. Using G*Power version 3.1.9.7 (Dusseldorf University, Dusseldorf, Germany) with a desired power of 0.80 and an alpha level of 0.05 for a two-tailed correlation test, the estimated required sample size was 85 participants.

To account for potential exclusions and ensure a sufficient sample size for robust analyses, we aimed to recruit a slightly larger sample. Given the extended data collection period, we collected data from eligible participants in the order they were encountered at the outpatient clinics until we reached a final sample size of 100, which was chosen for ease of analysis and reporting. This approach ensured a representative sample while adhering to the estimated sample size requirements based on the power analysis.

All patients were receiving mood stabilizers and/or antipsychotic pharmacotherapy at the time of the study. To ensure a standardized assessment of medication dosage, antipsychotic medications were recorded in chlorpromazine-equivalent dosages [27]. The most frequently prescribed mood stabilizers were lithium and valproate, while the antipsychotic treatments included quetiapine, aripiprazole, olanzapine, risperidone, paliperidone, chlorpromazine, and haloperidol.

Potentially eligible participants were identified from the pool of patients scheduled for appointments at the outpatient clinics the following day. Of the 225 patients initially identified, 55 were excluded due to the presence of psychiatric, neurological, or internal comorbidities. An additional 21 patients were excluded because they did not meet the symptomatic remission criteria. Eight patients were excluded due to invalid scale responses, and 10 patients declined to participate. Further exclusions were made for patients with current psychotic symptoms (n = 6), alcohol or substance abuse within the past five years (n = 10), a diagnosis of mania, hypomania, or major depressive episode (n = 10), prior electroconvulsive therapy (ECT) within the past six months (n = 5), pregnant or lactating women (n = 5), significant cognitive impairment (n = 5), and insufficient language proficiency (n = 5). The final sample consisted of 100 patients who met all inclusion criteria and completed the study assessments.

### 2.3 Measures

The following forms and scales were used to collect data for the study:

Sociodemographic and Clinical Data Form: It is a form prepared by the researchers for the purpose of the study and includes detailed evaluations regarding age, gender, educational status, profession, marital status, clinical diagnosis process and treatment to determine the sociodemographic characteristics of the participants.

HAM-D: A 17-item version of the depression rating scale, which was originally published in 1960 and was originally 21-item, was used frequently in the clinic. The items of the scale related to difficulty falling asleep, waking up at midnight, waking up early in the morning, somatic symptoms, genital symptoms, weight loss and insight were rated between 0–2, and the other items between 0–4. Maximum 53 points are scored. 0–7 points indicate no depression, 8–15 points indicate mild depression, 16–28 points moderate depression, 29 and above indicate severe depression. The validity and reliability of the scale has also been tested by Akdemir *et al*. (1996) [28].

Young Mania Rating Scale (YMRS): It is a scale filled in by the interviewer, prepared to measure the severity and change of the manic state. <13 points indicates remission. The scale consists of 11 items in total. There are subgroups such as elevated mood, increased movement and energy, sexual interest, sleep, irritability, speech rate and amount, thought disorder, thought content, destructive and aggressive behavior, external appearance, and insight. Its validity and reliability study in our country was conducted by Karadağ *et al*. [29].

Hamilton Anxiety Rating Scale (HAM-A): It is a scale published in 1959 and prepared to measure the severity of anxiety and filled in by the interviewer. The scale consists of 14 items. Each item is scored between 0 (not available) and 4 (severe). There are subgroups such as anxious temperament, tension, fears, insomnia, cognition, depressive temperament, muscular somatic symptoms, emotional somatic symptoms, cardiovascular symptoms, respiratory symptoms, gastrointestinal symptoms, genitourinary symptoms, autonomic symptoms, and behavior during the interview. Turkish validity and reliability study has been done [30].

Mindful Attention Awareness Scale (MAAS): It was developed by Brown and Ryan in 2003 [11]. It is a 15-item scale that measures the general tendency to be aware of and be mindful of instant experiences in daily life. Higher scores on the scale indicate high conscious awareness. It is a 6-point Likert-type scale (Almost always, most of the time, sometimes, rarely, quite rarely, almost never). Its validity and reliability study in our country was carried out by Özyeşil *et al*. [31].

Clinical Global Impression Scale (CGI): It is a scale developed by Guy and used by the observer to evaluate the severity of mental disorders or improvement in symptoms. Based on the observer’s general experience of the disease, the severity of the disease or the degree of improvement is graded between Not ill = 0 and Very Severely ill = 7 [32].

General Assessment of Functionality Scale (GAF): It is a scale that helps to monitor the clinical progress of individuals with its general framework, using a single measure. It is a measurement tool that evaluates a person’s psychological, social and professional functionality. Functionality impairments due to physical or environmental constraints cannot be evaluated separately. The evaluation made with the scale is made by grading the functionality of the person by giving a score between 1 and 100 by the clinician for a period in that time or in the past. High scores on the scale indicate high functionality [33].

### 2.4 Statistical Analysis

All statistical analyses were performed using IBM SPSS version 21.0 (IBM Corp., Armonk, NY, USA). Descriptive statistics were reported as means and standard deviations (SD) for normally distributed continuous variables and as medians with minimum and maximum values for non-normally distributed continuous variables. Categorical variables were summarized as frequencies and percentages. The normality of continuous variables was assessed using the Shapiro-Wilk test. Depending on the distribution, group comparisons were performed using the independent samples *t*-test, Mann-Whitney U test, or Kruskal-Wallis test. For ordinal data, if the normality assumptions were not met, we applied the Kruskal-Wallis test for comparisons involving more than two groups and the Mann-Whitney U test for two-group comparisons. To analyze correlations between continuous variables, we used Pearson correlation for normally distributed data and Spearman correlation for non-normally distributed data. Spearman correlation analysis was conducted to examine the relationships between MAAS scores and clinical variables, including HAM-D, HAM-A, CGI, and Functionality scores. To control for Type I errors due to multiple comparisons, a Bonferroni correction was applied, setting the corrected significance threshold at *p* < 0.0125 (0.05/4).

Following correlation analysis, univariate regression analyses were conducted for each significant variable. Those that remained significant in univariate analyses were then included in a multiple regression model to determine their independent contributions to mindful attention awareness. The final regression model identified HAM-D and HAM-A as significant independent predictors, while CGI and Functionality scores did not retain statistical significance and were excluded from the final model. To assess whether the sample size was sufficient to detect meaningful effects, a post-hoc power analysis was conducted. The results indicated that the study had high power (1–β >0.80) to detect significant correlations between MAAS and HAM-D/HAM-A, confirming the robustness of these findings. A *p*-value of <0.05 was considered statistically significant for all analyses except for the correlation analyses, for which a Bonferroni-corrected significance level of *p* < 0.0125 was used.

No missing data was detected. The study was prepared according to the STROBE-Statement guideline recommended for cross-sectional studies.

## 3. Results

The sociodemographic and clinical characteristics of the sample are shown in Table 1. There were no significant differences in MAAS scores between groups based on gender (*p* = 0.608), marital status (*p* = 0.496), having children (*p* = 0.699), educational status (*p* = 0.217), employment status (*p* = 0.310), medical comorbidities (*p *= 0.287), smoking status (*p* = 0.311), alcohol use (*p* = 0.684), suicide attempt history (*p* = 0.426), family history of psychiatric illness (*p* = 0.803), or electroconvulsive therapy history (*p* = 0.390). Similarly, there were no significant correlations between MAAS scores and age (*p* = 0.397), age of onset of disease (*p* = 0.847), age at diagnosis of disease (*p* = 0.881), time between onset of illness and diagnosis (*p* = 0.974), duration of illness (*p* = 0.098), chlorpromazine equivalent dosage (*p *= 0.497), type of mood stabilizers used (*p* = 0.056), lithium blood level (*p* = 0.202), or valproic acid blood level (*p* = 0.080). A low positive correlation was found between the number of hospitalizations and MAAS scores (*p* = 0.029).

**Table 1.** **Sociodemographic and clinical characteristics of patients with bipolar I disorder**.

Variable		Mean ± SD or Median (Min–Max)	n (%)	MAAS	*p*
Age (years)		39.1 ± 10.8		66.27 ± 11.99	0.397
Gender					0.608
	Female		55 (55.0)	65.71 ± 12.25	
	Male		45 (45.0)	66.96 ± 11.78	
Marital status					0.496
	Married		48 (48.0)	67.13 ± 13.29	
	Single/divorced/widowed		52 (52.0)	65.48 ± 10.74	
Having children					0.699
	Yes		47 (47.0)	66.77 ± 13.19	
	No		53 (53.0)	65.83 ± 10.94	
Educational status					0.217
	No formal education		2 (2.0)	71.00 ± 2.82	
	Primary school		30 (30.0)	63.13 ± 14.13	
	Secondary school		9 (9.0)	74.33 ± 9.07	
	High school		31 (31.0)	66.26 ± 11.19	
	University		28 (28.0)	66.71 ± 10.73	
Employment status					0.310
	Working		34 (34.0)	64.82 ± 12.08	
	Unemployed		61 (61.0)	67.30 ± 12.36	
	Student		5 (5.0)	63.60 ± 5.08	
Medical comorbidities					0.287
	No		68	67.13 ± 12.59	
	Yes		32	64.44 ± 10.60	
Hypertension			10 (10.0)		
Diabetes			9 (9.0)		
Hypothyroidism			8 (8.0)		
Hyperlipidemia			4 (4.0)		
Coronary artery disease			2 (2.0)		
Pulmonary diseases			3 (3.0)		
Gastroesophageal reflux disease			4 (4.0)		
Smoking					0.311
	Yes		42 (42.0)	64.83 ± 11.35	
	No		58 (58.0)	67.31 ± 12.44	
Alcohol use					0.684
	Yes		6 (6.0)	65.83 ± 2.41	
	No		94 (94.0)	66.30 ± 12.30	
Suicide attempt					0.426
	Yes		21 (21.0)	64.90 ± 12.10	
	No		79 (79.0)	66.63 ± 12.02	
Family history of psychiatric ıllness					0.803
	Yes		48 (48.0)	66.58 ± 11.22	
	No		52 (52.0)	65.98 ± 12.78	
Age of onset of disease (years)		24.9 ± 9.07		66.27 ± 11.99	0.847
Age at diagnosis of disease (years)		27.2 ± 9.12		66.27 ± 11.99	0.881
Time between onset of illness and diagnosis (Years)		0 (0–29)		66.27 ± 11.99	0.974
Duration of ıllness (Years)		11 (1–38)		66.27 ± 11.99	0.098
Number of hospitalizations		1.5 (0–22)		66.27 ± 11.99	0.029
Chlorpromazine equivalent dosage		500 (0–2200)		66.27 ± 11.99	0.497
Electroconvulsive therapy history					0.390
	Yes		39 (39.0)	64.97 ± 10.24	
	No		61 (61.0)	67.10 ± 13.00	
Mood stabilizers					0.056
	Lithium (Li)		57 (57.0)	66.54 ± 12.12	
	Valproic acid (VPA)		30 (30.0)	68.80 ±10.47	
	Combination therapy (Li + VPA)		11 (11.0)	58.36 ± 9.47	
	Neither lithium nor valproate		2 (2.0)	64.00 ± 32.53	
Lithium blood level		0.61 ± 0.18			0.202
Valproic acid blood level		64.59 ± 17.53			0.080

MAAS, Mindful Attention Awareness Scale; The *p*-values indicate statistical comparisons between subgroups regarding MAAS scores.

Table 2 presents the Spearman’s correlation coefficients between the MAAS and other clinical scales. As hypothesized, there were significant negative correlations between MAAS scores and both the HAM-D (r = –0.444, *p* < 0.001) and the HAM-A (r = –0.472, *p* < 0.001), indicating that higher mindful attention awareness was associated with lower levels of depression and anxiety symptoms. No significant correlation was found between MAAS scores and the Young Mania Rating Scale (YMS) (r = –0.141, *p* = 0.161). Additionally, MAAS scores were positively correlated with the Global Assessment of Functionality Scale (GAF) (r = 0.217, *p* = 0.030), suggesting that higher mindful attention awareness was associated with better overall functioning.

**Table 2.** **Correlations between Mindful Attention Awareness Scale (MAAS), Hamilton Depression Rating Scale (HAM-D), Young Mania Rating Scale (YMS), Hamilton Anxiety Rating Scale (HAM-A), Clinical Global Impression Scale (CGI), and Global Assessment of Functionality Scale (GAF) scores**.

		HAM-D	YMS	HAM-A	MAAS	CGI	GAF
HAM-D	r	1.000					
	*p*						
YMS	r	–0.001	1.000				
	*p*	0.992					
HAM-A	r	0.613**	0.028	1.000			
	*p*	<0.001	0.782				
MAAS	r	–0.444**	–0.141	–0.472**	1.000		
	*p*	<0.001	0.161	<0.001			
CGI	r	0.454**	0.177	0.226*	–0.208*	1.000	
	*p*	<0.001	0.078	0.024	0.038		
GAF	r	–0.552**	–0.271**	–0.420**	0.217*	–0.636**	1.000
	*p*	<0.001	0.006	<0.001	0.030	<0.001	

* *p* < 0.05, ** *p* < 0.01 (also denotes *p* < 0.0125 as per Bonferroni correction). r indicates Spearman’s correlation coefficients.

Table 3 presents the results of univariate and multivariable linear regression analyses examining the associations between the MAAS and various clinical scales. The multivariable model, which included the HAM-D, HAM-A, CGI, and GAF, explained 21.8% of the variance in MAAS scores (F(4,95) = 7.904, *p* < 0.001). In the multivariable analysis, HAM-D and HAM-A were significant independent predictors of MAAS scores. Specifically, both HAM-D and HAM-A demonstrated significant negative associations with MAAS, indicating that higher levels of depression and anxiety symptoms were associated with lower mindful attention awareness. Notably, while CGI and GAF were significant predictors of MAAS in the univariate analyses, they did not retain significance in the multivariable model, suggesting that their associations with MAAS may be explained by their relationships with HAM-D and HAM-A.

**Table 3.** **Univariate and multivariable regression analyses of factors associated with mindful attention awareness among patients with bipolar I disorder**.

Univariate regression analysis	Unstandardized		Lower	Upper	R^2^	*p* value	
	B	SE					
HAM-D	–1.561	0.301	–2.159	–0.963	0.215	<0.001	
HAM-A	–1.301	0.285	–1.866	–0.736	0.176	<0.001	
CGI	–2.089	1.005	–4.084	–0.094	0.042	0.040	
GAF	0.254	0.122	0.011	0.496	0.042	0.041	
Multivariable regression analysis	B	SE	Lower	Upper	Adjusted R^2^	*p* value	*p*’ value
HAMD	–1.122	0.430	–1.976	–0.268	0.218	0.011	<0.001
HAM-A	–0.751	0.370	–1.486	–0.016		0.045	
CGI	–0.949	1.208	–3.347	1.448		0.434	
GAF	–0.153	0.154	–0.459	0.152		0.322	

B, unstandardized coefficient; SE, standard error; Lower and Upper values indicate the confidence interval. The adjusted R^2^ value represents the proportion of variance explained by the model. The *p*-values indicate statistical significance for each predictor, while *p*’ represents the overall significance of the regression model (ANOVA test).

## 4. Discussion

Our study revealed a significant association between mindful attention awareness and residual depressive and anxiety symptoms in patients with bipolar I disorder. We found that higher levels of mindful attention awareness were associated with lower levels of both depressive and anxiety symptoms, even during periods of clinical remission. This suggests that mindful attention awareness may act as a buffer against the persistence of residual mood symptoms, highlighting its potential importance in achieving complete remission.

Furthermore, our findings underscore the interconnectedness of residual depressive and anxiety symptoms in bipolar I disorder. Individuals with higher levels of residual depression also tended to experience more anxiety symptoms, emphasizing the complex interplay between these symptom domains. Additionally, we observed that higher levels of residual depressive, manic, and anxiety symptoms were associated with reduced functionality, highlighting the detrimental impact of these symptoms on patients’ daily lives.

While our regression model revealed a statistically significant relationship between residual depressive and anxiety symptoms and mindful attention awareness, it only explained 21.8% of the variance in MAAS scores. This indicates that other factors, such as personality traits, coping mechanisms, social support, and specific medication effects, may also contribute to mindful attention awareness in this population

While our model highlights the significant role of depression and anxiety in influencing mindful attention awareness, it also underscores the complex interplay of other factors that warrant further investigation. Our findings are consistent with previous research demonstrating that depression and anxiety symptoms often co-occur [34]. This is further supported by a recent study, which specifically examined the clinical characteristics and etiological factors of comorbid major depressive disorder (MDD) and social anxiety disorder (SAD) [35]. Similarly, our study revealed a strong association between residual depressive and anxiety symptoms in patients with bipolar I disorder. This interconnectedness underscores the complex nature of mood regulation in BD and highlights the need for treatments that address both depressive and anxiety symptoms.

When we look at mindfulness-based interventions in bipolar disorder, mindfulness-based cognitive therapy was found to be effective in reducing depressive symptoms and anxiety associated with bipolar disorder in most of the studies, and it was found that there was no change in mania scores [36].

When we consider mindful attention awareness as a personality trait, those who have this trait have a higher level of personal control, autonomy, executive functions, empathy, self-esteem, life satisfaction, personal efficacy perception, optimism, and positive affect [37]. Besides, mindfulness interventions have been found to be effective in psychiatric diseases such as recurrent major depression, bipolar disorder, anxiety disorders, eating disorders, substance use disorders, and psychological distress related to chronic pain, cancer, and chronic physical diseases [38, 39].

Our findings also highlight the detrimental impact of residual symptoms on functionality in patients with bipolar I disorder. We observed a significant negative correlation between residual depressive and anxiety symptoms and functionality scores, indicating that even subsyndromal levels of these symptoms can impair daily functioning and overall well-being. As residual symptoms are fundamentally related to the functionality and subjective well-being of patients, this underscores their importance as a key treatment target in bipolar I disorder [40]. They not only contribute to distress but also hinder patients’ ability to lead fulfilling lives.

Given the complex interplay between residual depressive and anxiety symptoms and their impact on functionality, it is essential to explore interventions that can effectively target these interconnected challenges. Mindfulness-based interventions, such as MBCT, have shown promise in addressing both mood symptoms and functional impairments in individuals with bipolar disorder. With MBCT, there is an increase in mindful attention awareness, a decrease in depressive symptoms, emotion regulation, psychological well-being, positive affect, and psychosocial functionality [36]. With MBCT, depressive symptoms and suicidal thoughts, and to a lesser extent, mania and anxiety symptoms decrease.

One potential mechanistic hypothesis underlying the therapeutic effects of mindfulness interventions in BD involves the modulation of activity within the limbic network, a complex brain region critically implicated in emotional regulation. Extensive research has demonstrated that individuals with BD often exhibit hyperactivity within limbic structures, particularly the amygdala, which is associated with heightened emotional reactivity and instability [41, 42, 43]. This limbic hyperactivity is thought to contribute to the characteristic mood fluctuations and emotional dysregulation observed in BD.

Mindfulness-based interventions, such as MBCT, may exert their therapeutic effects by promoting emotion regulation strategies that directly target and modulate limbic activity. Neuroimaging studies have shown that mindfulness practice can lead to structural and functional changes in the brain, including increased gray matter density in regions associated with emotion regulation, such as the anterior cingulate cortex and insula [43, 44, 45]. Moreover, functional MRI studies have demonstrated that mindfulness practice can attenuate amygdala reactivity in response to emotional stimuli, suggesting a direct influence on limbic activity [41, 43].

By enhancing emotion regulation abilities and reducing limbic hyperactivity, mindfulness-based interventions may provide a means of interrupting the cycle of emotional reactivity and mood dysregulation that characterizes BD. This neural mechanism could explain how mindfulness practices, such as focused attention and non-judgmental awareness, contribute to the alleviation of residual mood symptoms and the promotion of overall well-being in individuals with BD. Further research is needed to fully elucidate the specific neural pathways involved and to determine the optimal mindfulness-based interventions for targeting limbic dysfunction in BD.

In a study in which bipolar I and II patients were included, DBT skills training, mindful attention awareness techniques training, and general bipolar disorder psychoeducation were given to the patients for 12 weeks, 90 minutes per week, and an increase in mindful attention awareness, a decrease in depressive symptoms, and a tendency to decrease in emergency service admissions were found [36]. In the study conducted by Ives-Deliperi *et al*. [46] including healthy and patient control groups, an increase was detected in the medial prefrontal cortex and posterior parietal lobe in bipolar patients who underwent MBCT for 8 weeks. They found a strong correlation between increased signal in the medial prefrontal cortex and increased mindful attention awareness [46]. Contrary to the aforementioned studies, in the study of Perich *et al*. [23], in which 95 bipolar patients were included, 48 of the patients were administered additional MBCT to the current treatment, in a 12-month follow-up, they reported that there was no difference in terms of time to relapse, total number of episodes and severity of mood symptoms. In the same study, a decrease in anxiety symptoms was found in the MBCT group.

It is important to acknowledge that our study did not explicitly measure or control for several potential contributing factors that could influence both mindful attention awareness and residual mood symptoms. For instance, concurrent psychotherapy, particularly therapies that incorporate mindfulness techniques or emphasize emotional regulation, could contribute to increased mindful attention awareness and reduced residual symptoms [12]. Similarly, social support networks can play a crucial role in mitigating stress and promoting emotional well-being, which could, in turn, influence both mindful attention awareness and residual mood symptoms [47]. Furthermore, individual differences in personality traits, such as neuroticism, resilience, and openness to experience, might influence both the propensity to engage in mindful attention and the experience of residual mood symptoms [48]. Coping mechanisms, such as adaptive or maladaptive strategies for managing stress and emotional challenges, could also play a role. Additionally, lifestyle factors, such as exercise, sleep, and diet, may influence both mindful attention awareness and mood regulation [49]. It is also possible that specific medication effects, beyond those we measured, could contribute to variations in mindful attention awareness and residual symptoms [36].

Exploring these potential contributing factors through in-depth qualitative or mixed-methods studies could provide valuable insights into the complex interplay between mindful attention awareness, residual mood symptoms, and functionality in individuals with bipolar I disorder. Understanding these nuanced relationships could inform the development of more targeted and personalized interventions to enhance mindful attention awareness and improve overall well-being in this population.

Our findings highlight the potential of mindfulness-based interventions to address residual depressive and anxiety symptoms in patients with bipolar I disorder. Integrating these interventions into clinical practice could offer valuable tools for enhancing emotional regulation and improving overall well-being.

Specifically, MBCT may be particularly beneficial for this population [46]. Given its demonstrated efficacy in reducing depressive symptoms in BD, MBCT could help patients develop non-judgmental awareness of residual symptoms, thereby reducing emotional reactivity and improving coping strategies. Additionally, techniques from Mindfulness-Based Stress Reduction (MBSR), such as breathing exercises, body scans, and mindful movement, could be integrated into routine psychiatric care to enhance patients’ ability to manage stress and regulate emotions [22]. However, it is important to tailor mindfulness approaches for the unique needs of BD patients. The study suggests that mindfulness may be less effective or even counterproductive in manic states, as it could potentially amplify racing thoughts or increase agitation [24]. Therefore, interventions should be carefully tailored to avoid excessive focus on self-reflection during hypomanic or manic episodes. Instead, practices that emphasize grounding and present-moment awareness, such as mindful walking or sensory awareness exercises, might be more appropriate during these phases.

By thoughtfully integrating and adapting mindfulness-based interventions, clinicians can provide patients with valuable skills for managing residual symptoms, improving emotional regulation, and enhancing their overall quality of life.

## 5. Limitations

This study has several limitations. First, the cross-sectional design precludes the establishment of causality between mindful attention awareness and residual mood symptoms in bipolar I disorder. While we observed significant associations, we cannot definitively conclude whether lower mindful attention awareness contributes to increased residual symptoms or whether residual symptoms lead to reduced mindfulness. Longitudinal studies are needed to elucidate causal relationships and the temporal dynamics between these variables. Second, the modest sample size and single-institution setting may limit the generalizability of our findings to other populations with bipolar I disorder. A larger multi-center study with a more diverse sample would enhance the external validity of these results. Third, differences in symptom severity must be considered when interpreting our findings. Our sample consisted of remitted BD-I patients; therefore, these findings may not fully apply to individuals experiencing active mood symptoms. Future research should explore how mindfulness interacts with mood symptoms at different phases of the illness, including during depressive and manic episodes. Fourth, cultural factors may influence mindful attention awareness. Since mindfulness is shaped by cultural attitudes toward mental health and emotional regulation, our findings may require further validation in populations with different cultural backgrounds or treatment settings. Future cross-cultural studies could help determine whether these relationships hold across diverse patient groups. Fifth, reliance on self-reported measures, such as the MAAS, introduces potential biases related to subjective interpretation and recall. Although these scales are widely used and validated, they may not fully capture the objective experience of mindful attention awareness. Future studies could incorporate objective measures, such as behavioral tasks or physiological indicators, to complement self-report data and provide a more comprehensive assessment. Sixth, while we collected data on lithium and valproic acid levels, the influence of other medications on symptoms and mindfulness was not assessed. Given the complex interplay between medications, mood, and cognition in BD-I, future studies should examine the specific impact of various psychotropic medications on mindful attention awareness and residual symptoms. Seventh, the lack of evaluation of psychotherapy or mindfulness-based interventions limits insight into the modifiability of mindful attention awareness. Future research should examine whether targeted interventions, such as MBCT, can enhance mindful attention awareness and subsequently reduce residual mood symptoms in this population.

Finally, the absence of a control group restricts our ability to determine whether the observed relationships are specific to bipolar I disorder. It is possible that similar associations between mindful attention awareness and residual mood symptoms exist in other psychiatric conditions or even in healthy individuals. Future studies incorporating control groups will help clarify the specificity of these findings to BD-I. Despite these limitations, our study provides valuable insights into the interplay between mindful attention awareness, residual mood symptoms, and functionality in bipolar I disorder. These findings have important implications for clinical practice and future research.

## 6. Future Research

Several avenues for future research emerge from our findings. First, longitudinal studies are needed to establish causality and examine the temporal dynamics between mindful attention awareness and residual mood symptoms. This would help clarify whether lower mindful attention awareness contributes to increased residual symptoms over time, or vice versa. Second, research with larger, more diverse samples would enhance the generalizability of our findings and provide a more nuanced understanding of the relationship between mindful attention awareness and residual symptoms across different subgroups of patients with bipolar I disorder. Third, incorporating objective measures, such as behavioral tasks or physiological indicators, alongside self-reported measures would provide a more comprehensive assessment of mindful attention awareness and its impact on mood regulation. Fourth, future studies should examine the influence of specific medications, psychotherapy, and other interventions on mindful attention awareness and residual symptoms. This would help identify factors that may enhance or hinder the development and maintenance of mindful attention awareness in this population. Finally, investigating the neural mechanisms underlying the relationship between mindful attention awareness and residual symptoms, particularly within the limbic network, could provide valuable insights into the therapeutic effects of mindfulness-based interventions. This could lead to the development of more targeted and effective treatments for bipolar I disorder.

## 7. Clinical Implications

Our findings suggest that mindful attention awareness may play a crucial role in mitigating residual depressive and anxiety symptoms in patients with bipolar I disorder. This has important clinical implications, as residual symptoms significantly impact functionality and quality of life, even during periods of remission. Clinicians should consider incorporating mindfulness-based interventions, such as MBCT, into treatment plans to help patients cultivate mindful attention awareness and manage residual symptoms. Moreover, our results highlight the interconnectedness of residual depressive and anxiety symptoms, suggesting that interventions should target both symptom domains. Integrating mindfulness practices with other evidence-based treatments, such as medication and psychotherapy, may provide a comprehensive approach to managing bipolar I disorder and improving overall well-being. Furthermore, clinicians should routinely assess mindful attention awareness and functionality in patients with bipolar I disorder, as these factors can provide valuable insights into treatment progress and guide personalized interventions. By recognizing the importance of mindful attention awareness and addressing residual symptoms, clinicians can help patients achieve more complete remission and improve their overall quality of life.

## 8. Conclusion

This study provides valuable insights into the relationship between mindful attention awareness and residual mood symptoms in bipolar I disorder. Our findings suggest that mindful attention awareness may protect against residual depressive and anxiety symptoms, which impair functionality and well-being. Although further research is needed to establish causality and explore the underlying mechanisms, our results support integrating mindfulness-based interventions into treatment protocols for bipolar I disorder. By addressing residual symptoms and promoting mindful attention awareness, clinicians can help patients achieve more comprehensive remission and improve their quality of life.

## Availability of Data and Materials

The data that support the findings of this study are available on request from the corresponding author.
